# Supplementary material for: High performance tandem organic solar cells via a strongly infrared-absorbing narrow bandgap acceptor
Source: Nat Commun. 2021 Jan 8;12:178. doi: 10.1038/s41467-020-20431-6 (PMC7794321; doi:10.1038/s41467-020-20431-6)
Supplement: Supplementary file 1 — Supplementary Information [file 41467_2020_20431_MOESM1_ESM.pdf]

SUPPLEMENTARY INFORMATION

High performance tandem organic solar cells *via* a strongly infrared-absorbing narrow bandgap acceptor

SUPPLEMENTARY FIGURES

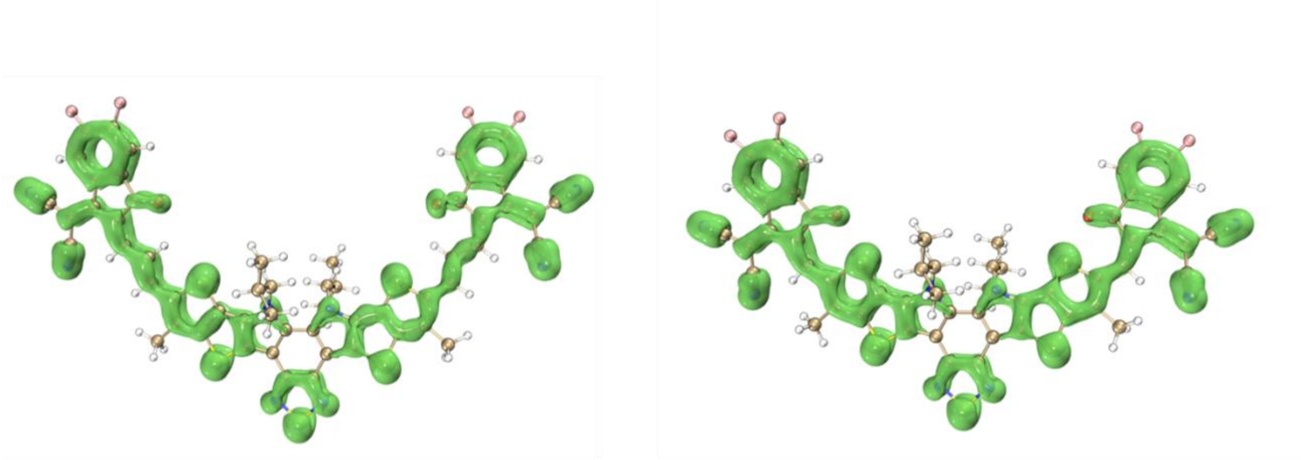

BTPV-4F **Y6**

Supplementary Fig. 1. Simulated localized orbital locator obtained by DFT calculations for simplified molecules of BTPV-4F and Y6.

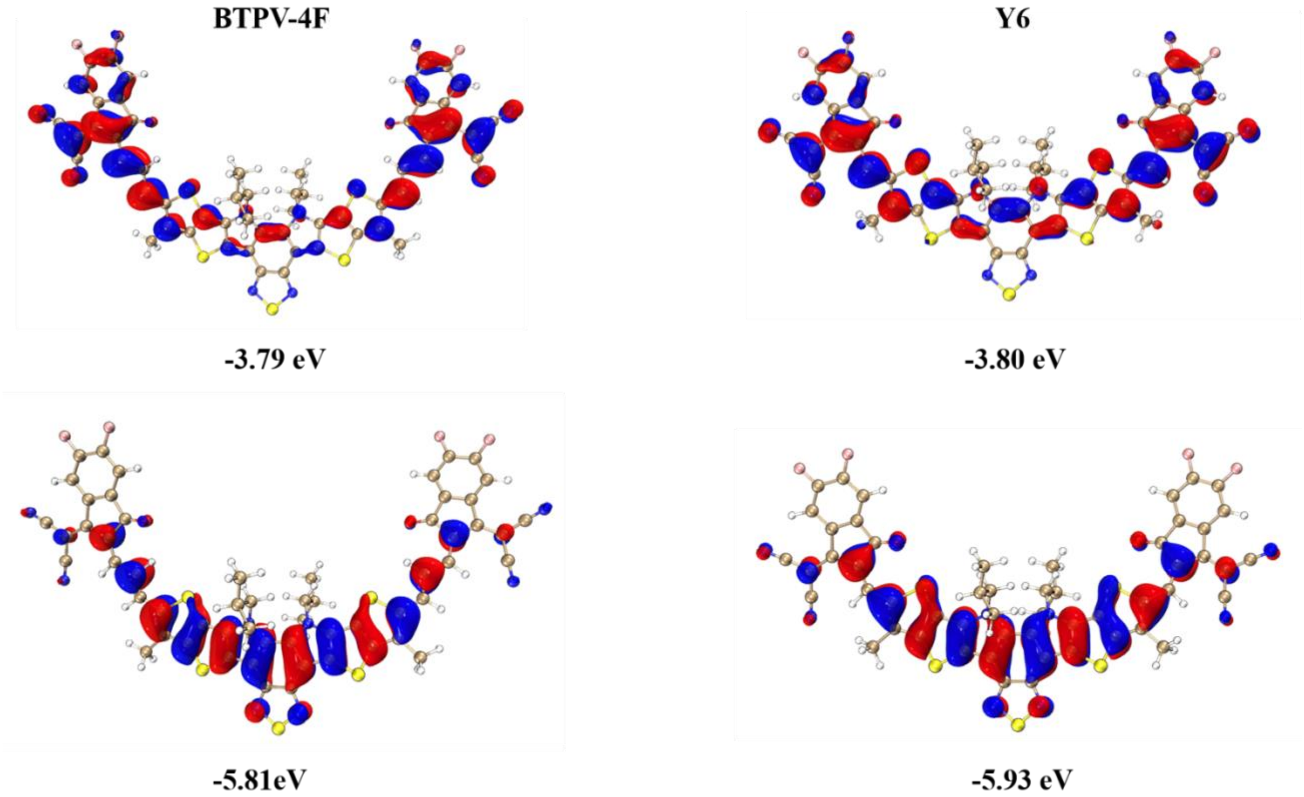

Supplementary Fig. 2. Simulated frontier molecular orbitals obtained by DFT calculations for simplified molecules of BTPV-4F and Y6.

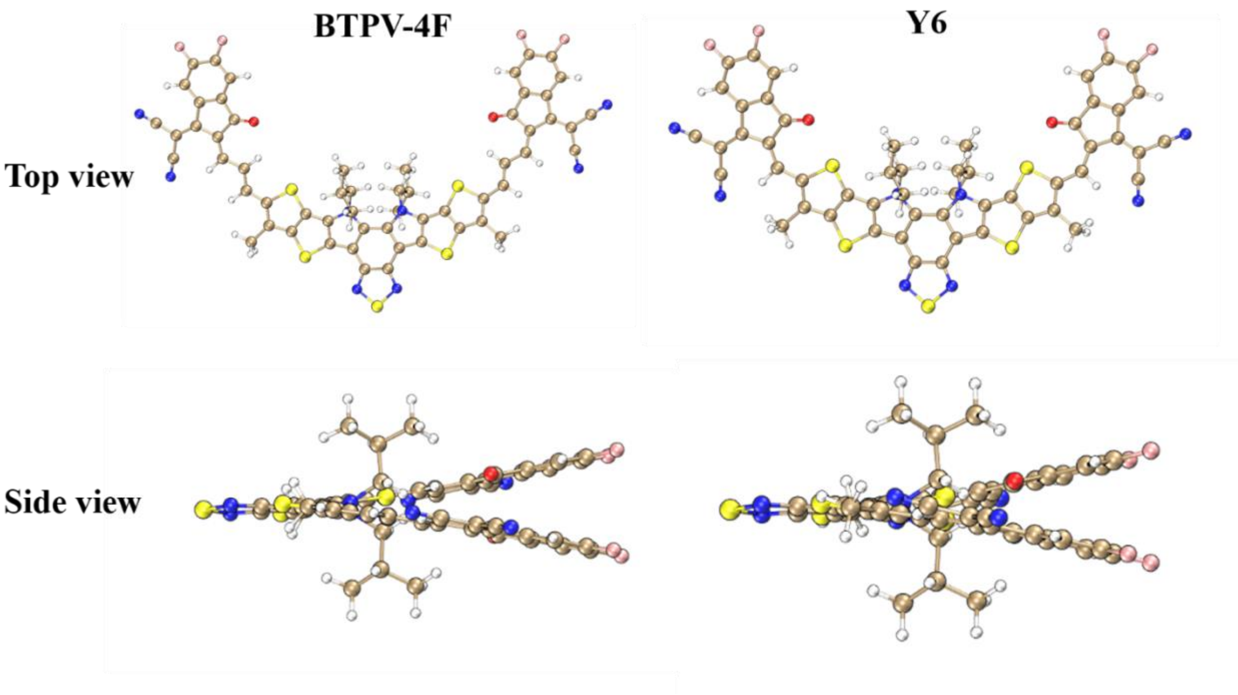

Supplementary Fig. 3. Simulated optimized geometries obtained by DFT calculations for simplified molecules of BTPV-4F and Y6.

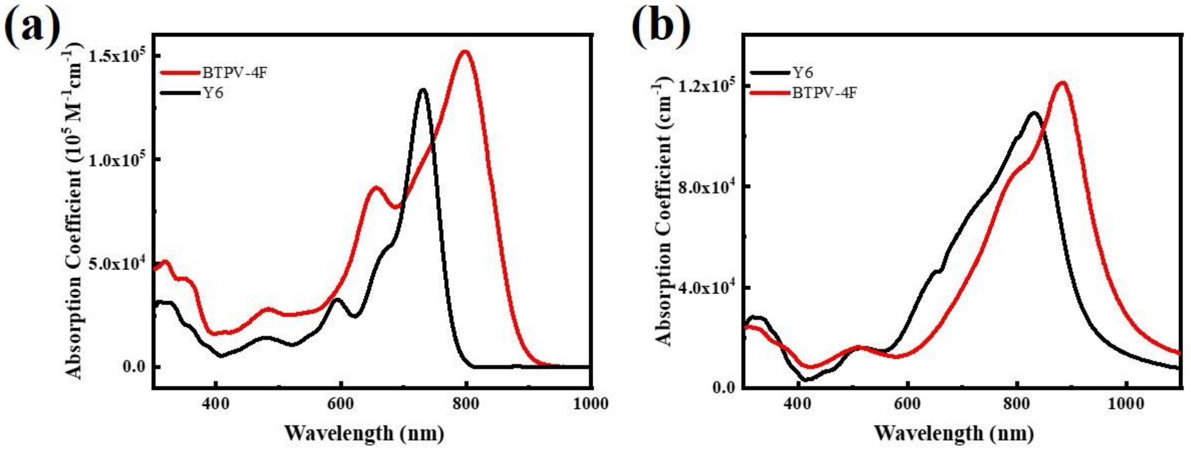

Supplementary Fig. 4 (a) Absorption spectra of BTPV-4F and Y6 in chloroform solution. (b) Absorption spectra of BTPV-4F and Y6 films.

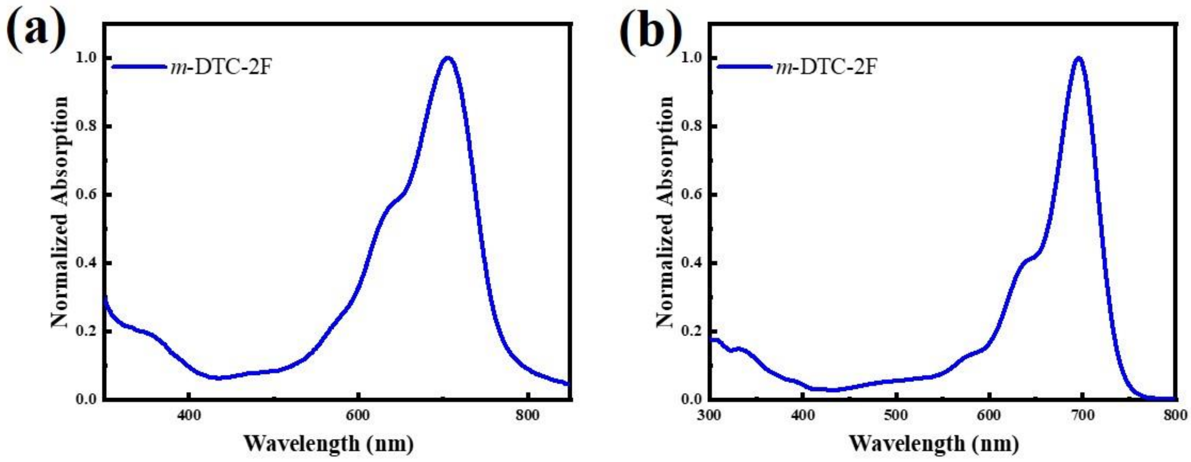

Supplementary Fig. 5. (a) Normalized absorption spectrum of the *m*-DTC-2F film. (b) Normalized absorption spectrum of the *m*-DTC-2F in chloroform solution.

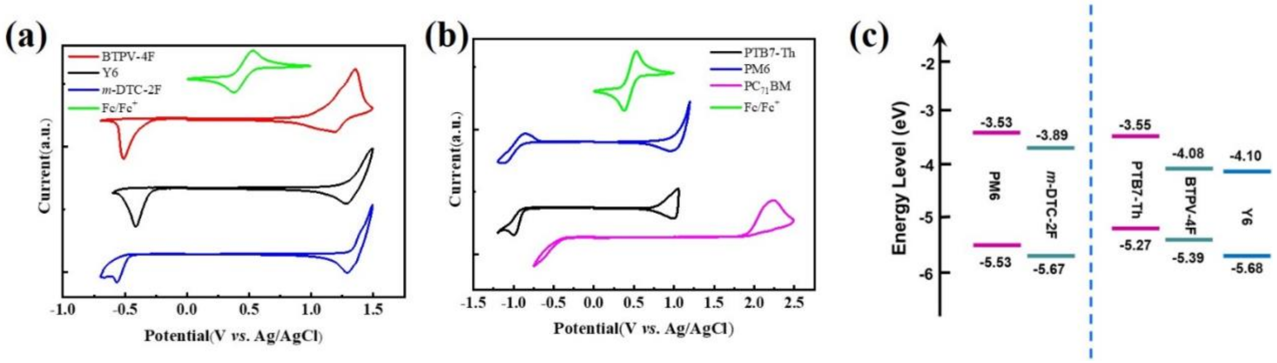

Supplementary Fig. 6. Cyclic voltammograms of (a) acceptors of BTPV-4F, Y6 and *m*-DTC-2F, and (b) donors of PTB7-Th and PM6 and acceptor $PC_{71}BM$. (c) Energy level diagram of the donor and acceptor.

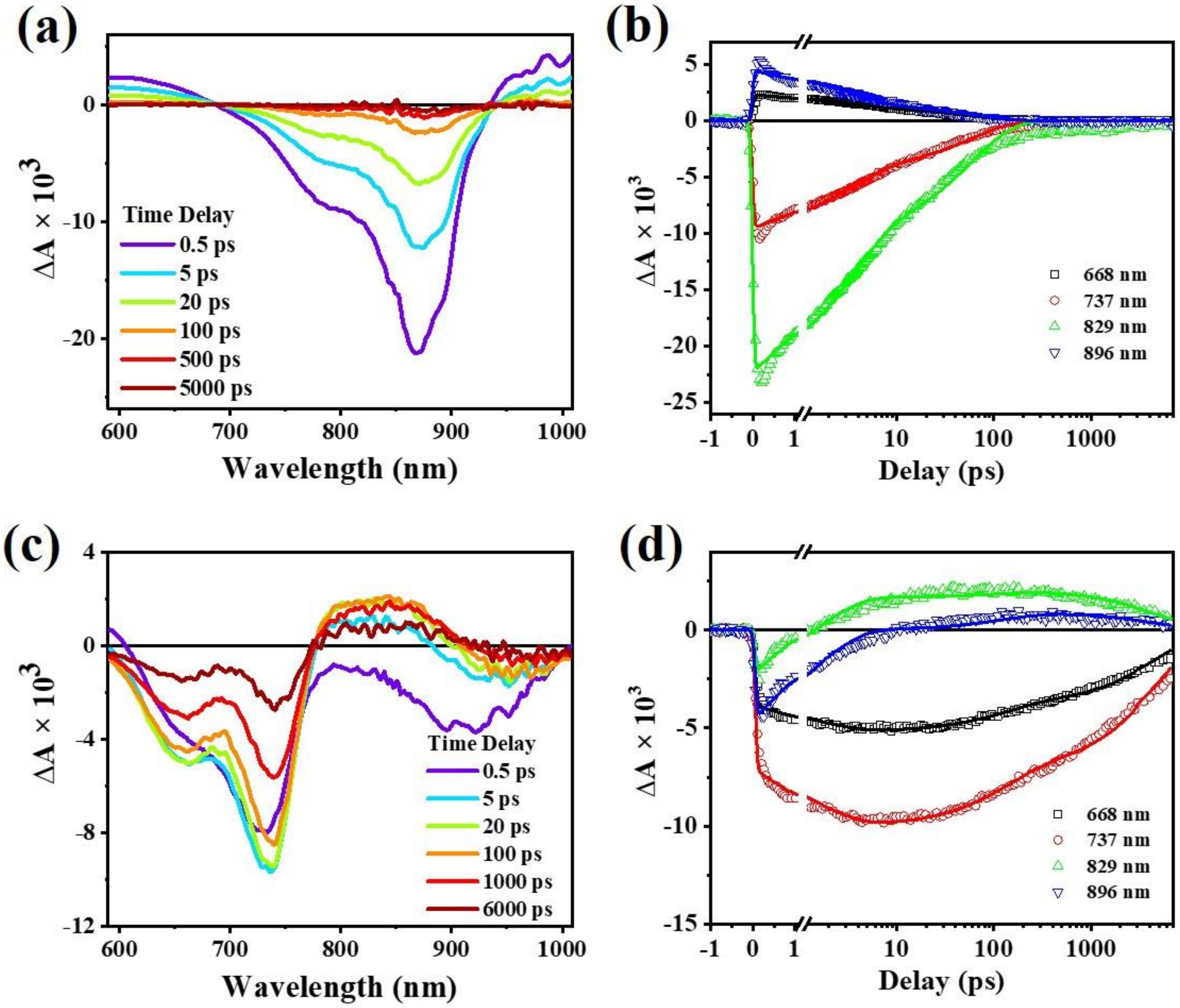

Supplementary Fig. 7 (a) Femtosecond transient absorption spectra of the pristine BTPV-4F film at selected time delays. (b) Transient kinetics of pristine BTPV-4F film at selected wavelengths with global fitting. (c) Femtosecond transient absorption spectra of PTB7-Th: BTPV-4F blend film at selected time delays. (d) Transient kinetics of pristine PTB7-Th: BTPV-4F blend film at selected wavelengths with global fitting.

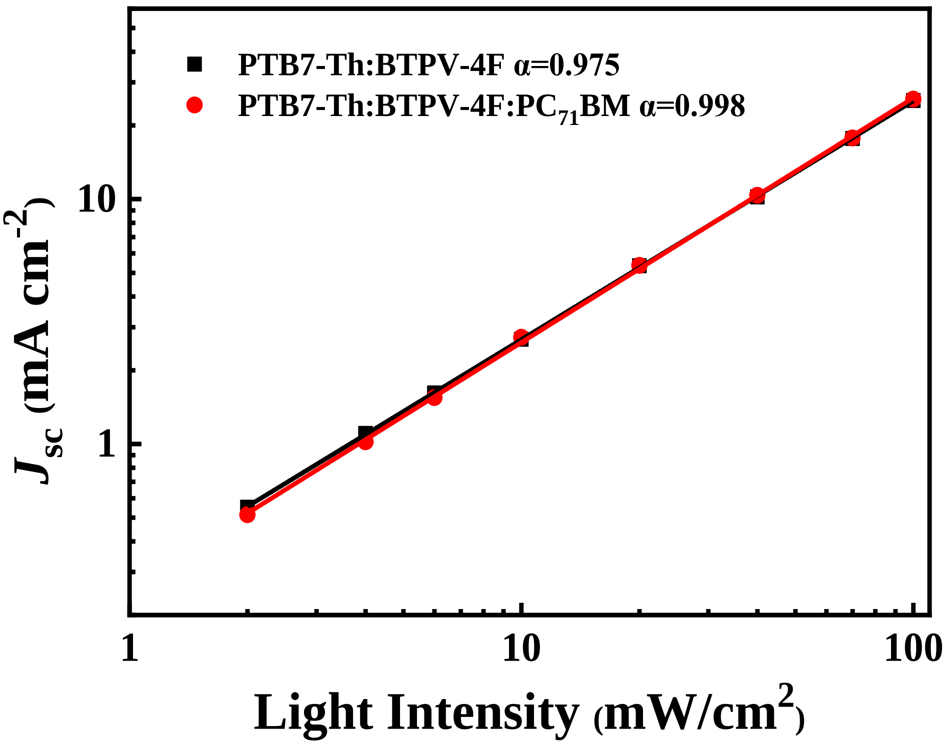

Supplementary Fig. 8. Dependence of J_{sc} of the OSCs *versus* the light intensity irradiated.

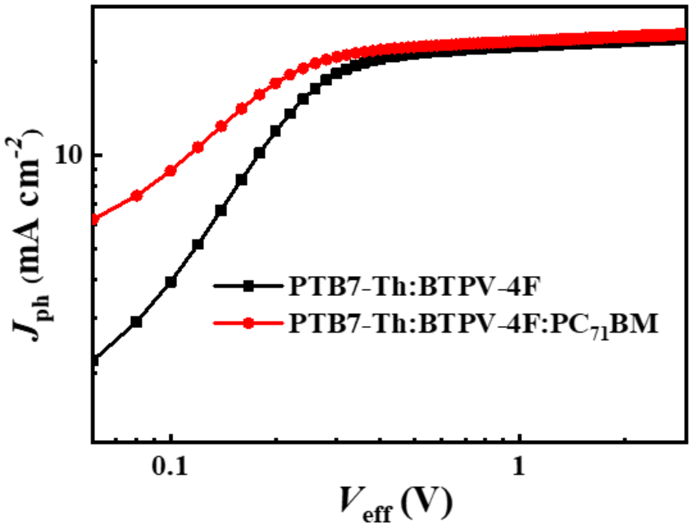

Supplementary Fig. 9. Plots of photocurrent density (J_{ph}) *versus* effective voltage (V_{eff}) of the binary and the ternary OSCs.

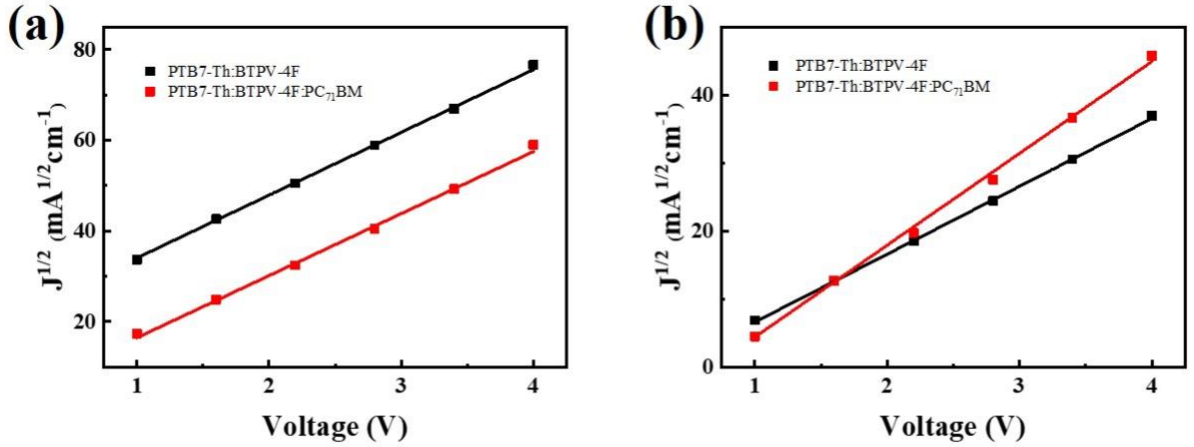

Supplementary Fig. 10. The mobilities measurement plots of (a) hole-only devices and (b) electron-only devices based on different blend active layers.

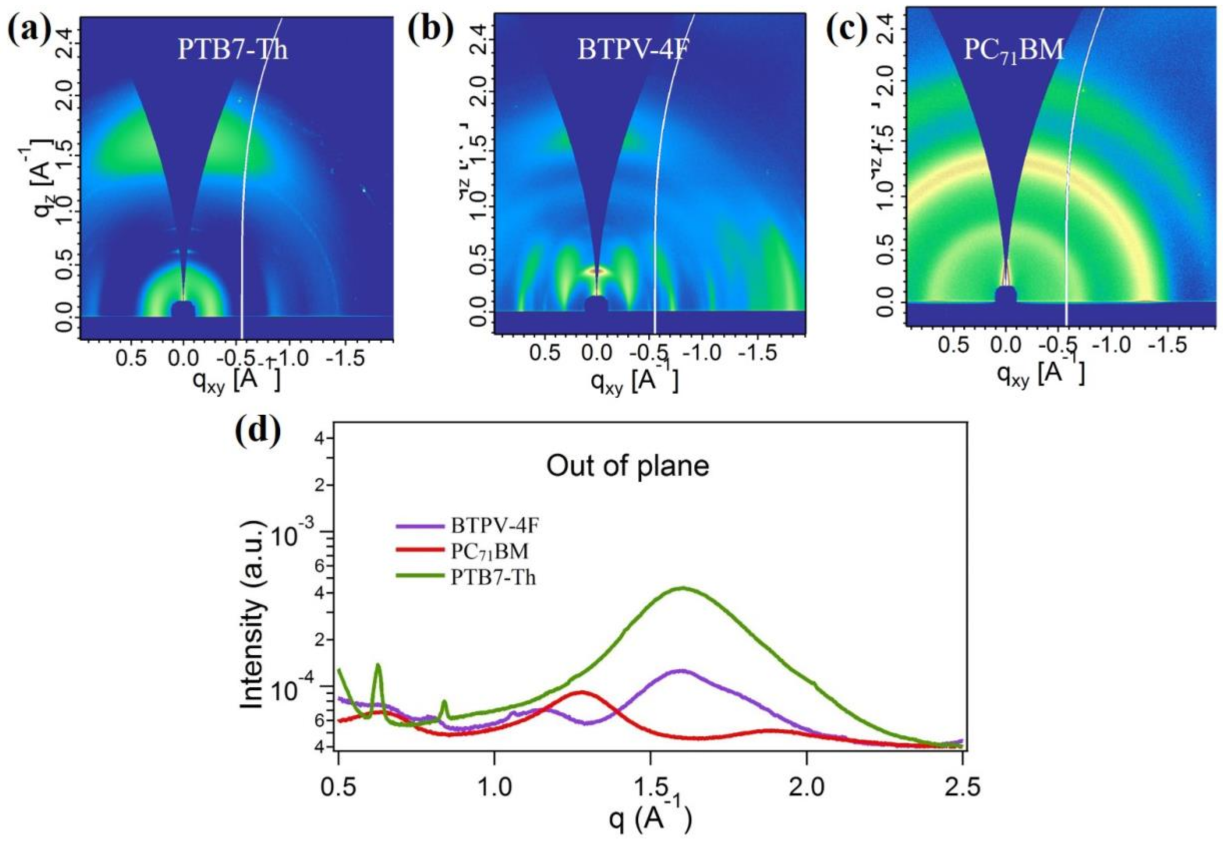

Supplementary Fig. 11. (a) GIWAXS images of PTB7-Th. (b) GIWAXS images of BTPV-4F. (c) GIWAXS images of $PC_{71}BM$. (d) Corresponding out-of-plane line cuts of the GIWAXS images of neat films of PTB7-Th, BTPV-4F and $PC_{71}BM$.

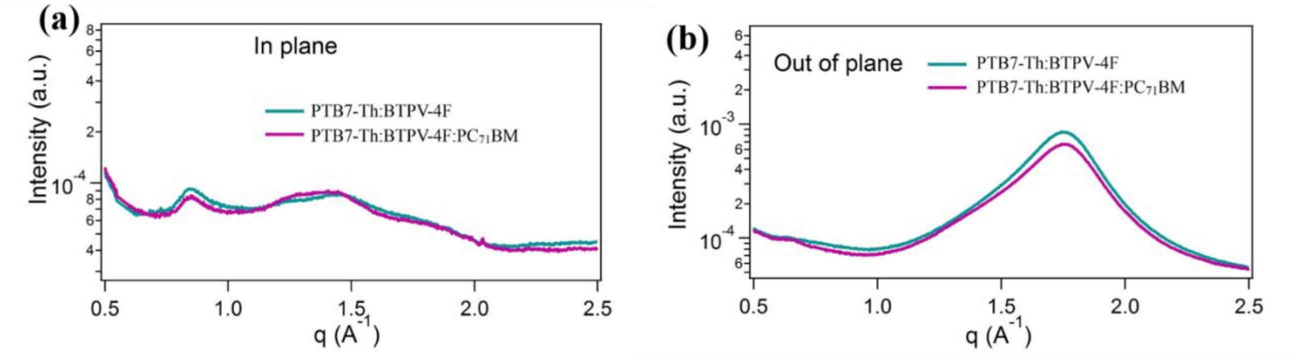

Supplementary Fig. 12. (a) In-plane line cuts of the GIWAXS images of blend films. (b) Out-of-plane line cuts of the GIWAXS images of blend films.

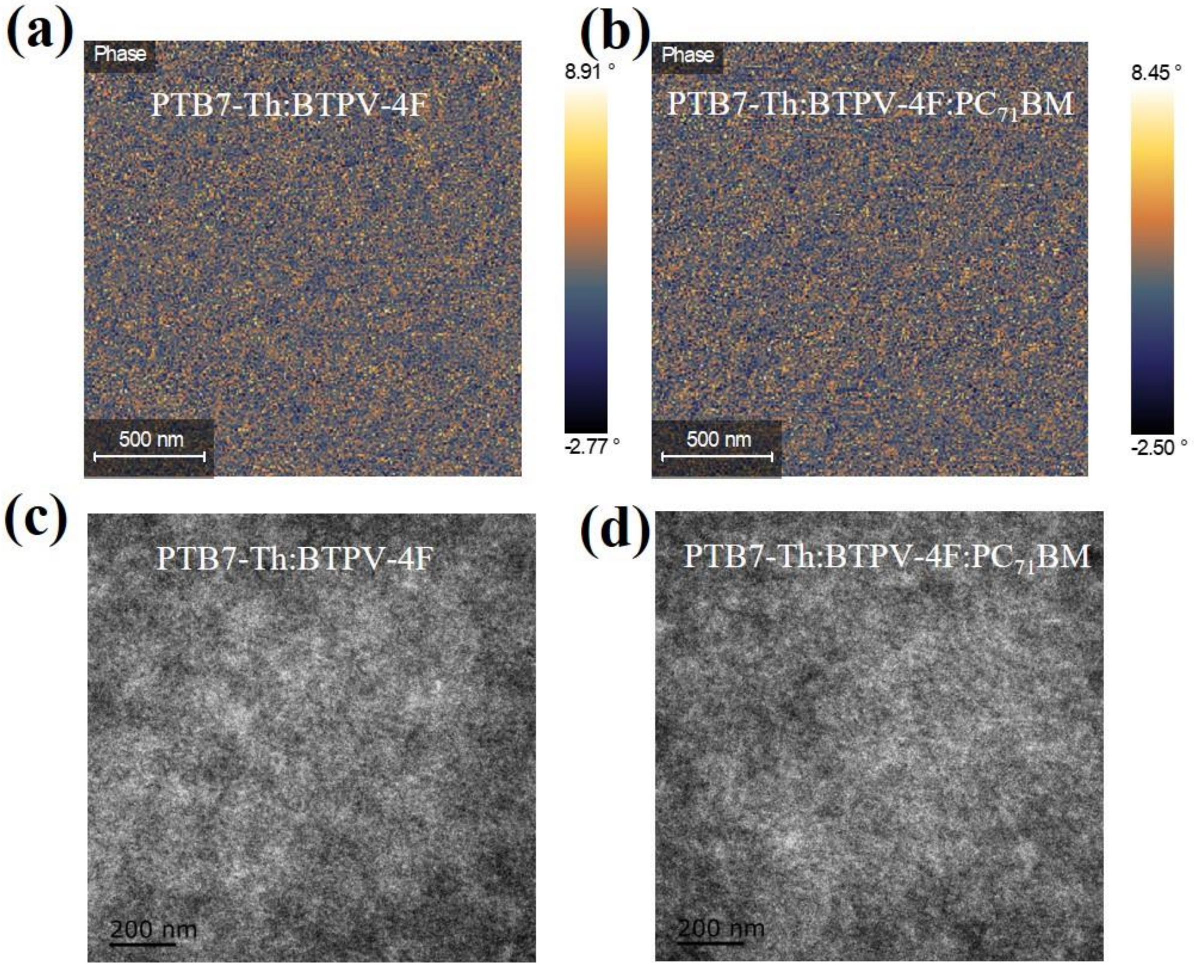

Supplementary Fig. 13. AFM phase images of (a) binary blend film of PTB7-Th:BTPV-4F and (b) ternary blend film of PTB7-Th:BTPV-4F:$PC_{71}BM$. TEM images of (c) PTB7-Th:BTPV-4F and (d) PTB7-Th:BTPV-4F:$PC_{71}BM$ films.

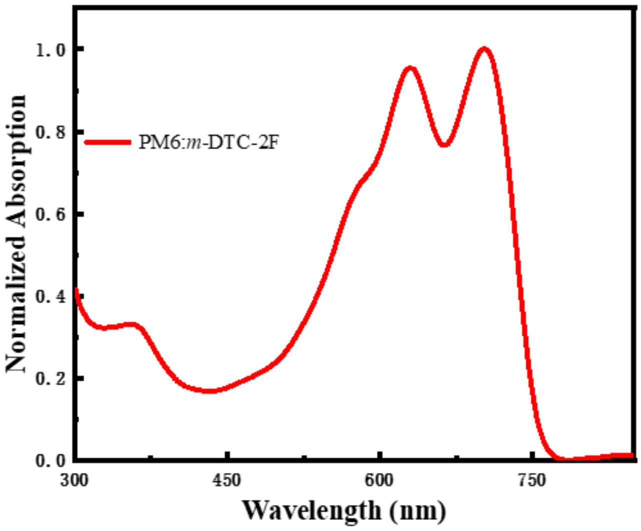

Supplementary Fig. 14. Film absorption spectrum of PM6:*m*-DTC-2F blend.

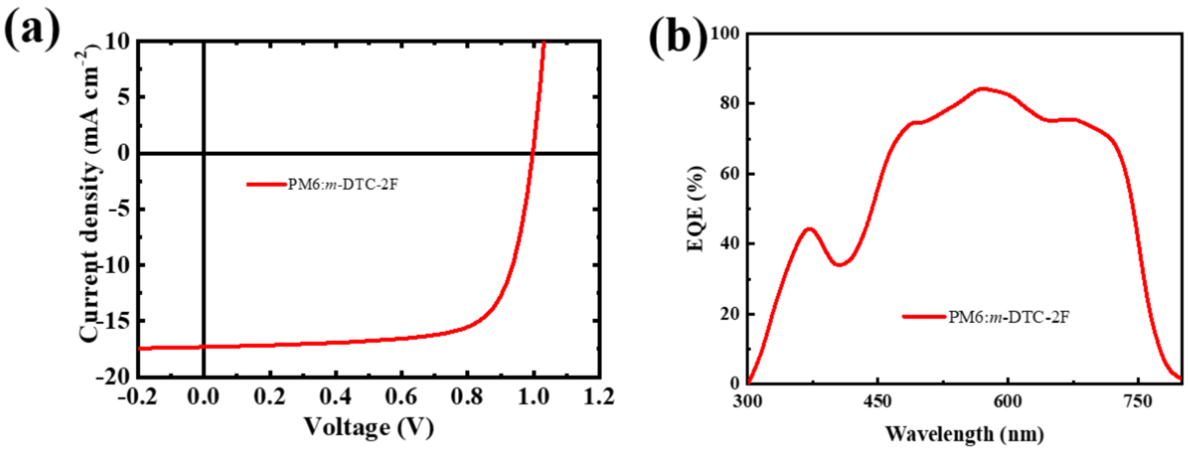

Supplementary Fig. 15. (a) The *J*-*V* curves of the OSCs based on PM6:*m*-DTC-2F with 100 nm active layer under the illumination of AM1.5G, 100 mW cm^{-2}. (b) EQE spectra of the corrsponding OSCs.

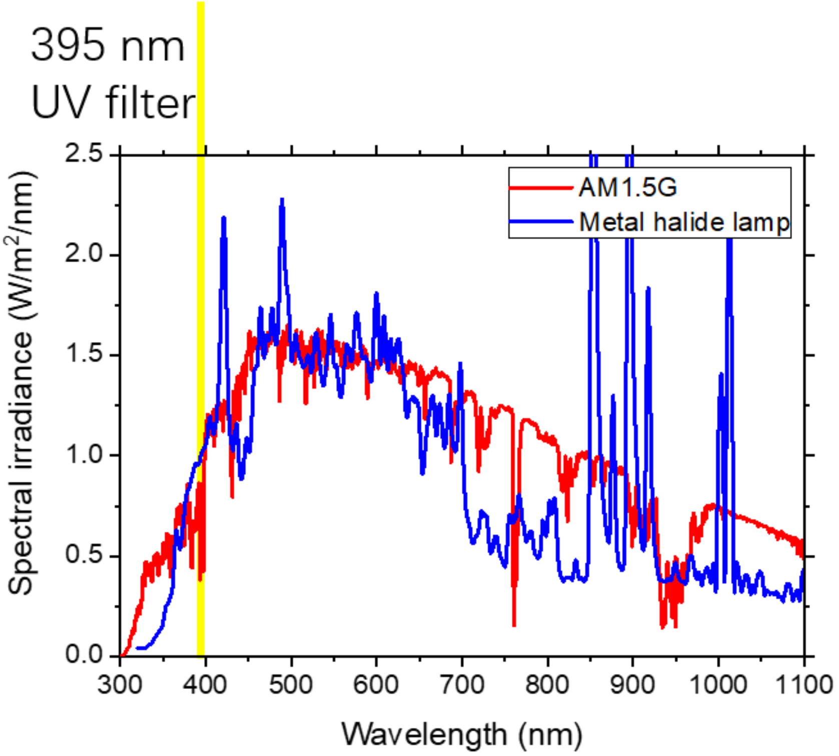

Supplementary Fig. 16. Illumination spectrum of light source used in stability test.

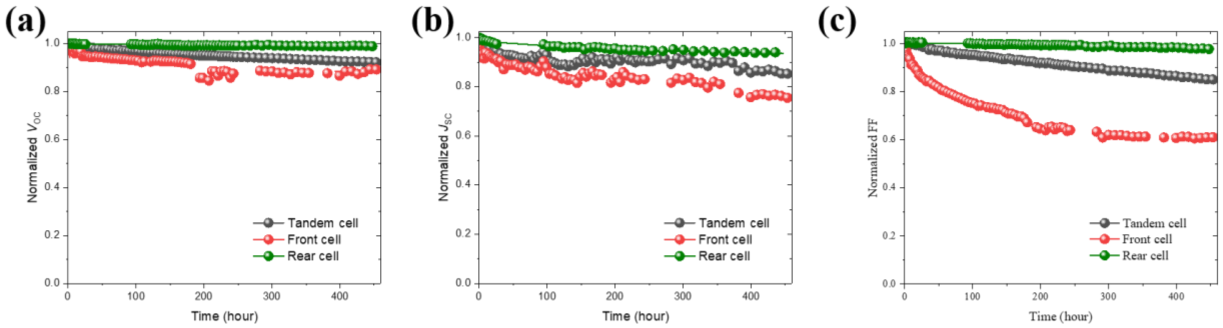

Supplementary Fig. 17. Evolution of (a) open-circuit voltage, (b) short-circuit current density and (c) fill factor in the test of photostability.

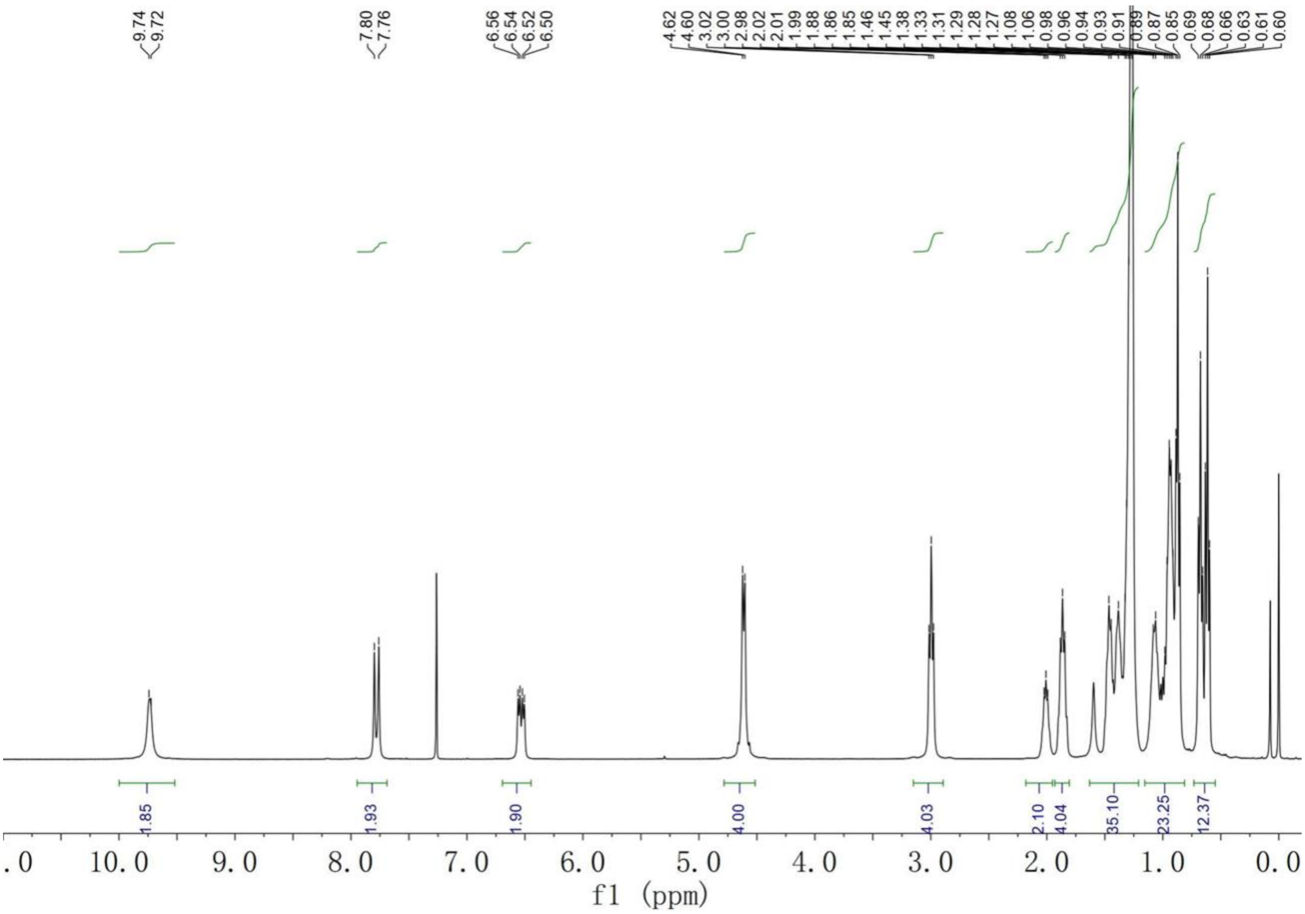

Supplementary Fig. 18. ^{1}HNMR spectra of BTPV-CHO.

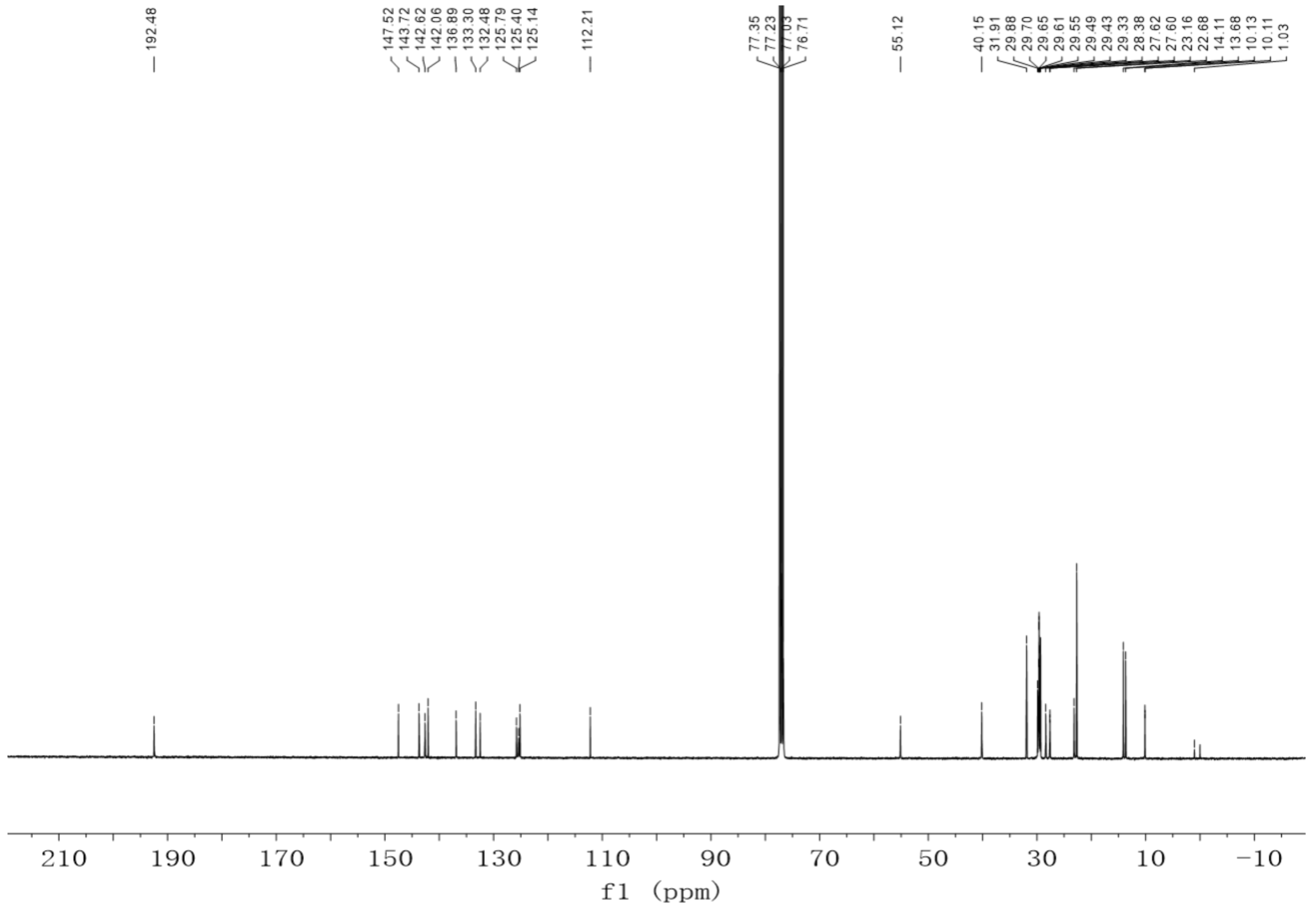

Supplementary Fig. 19. ^{13}CNMR spectra of BTPV-CHO.

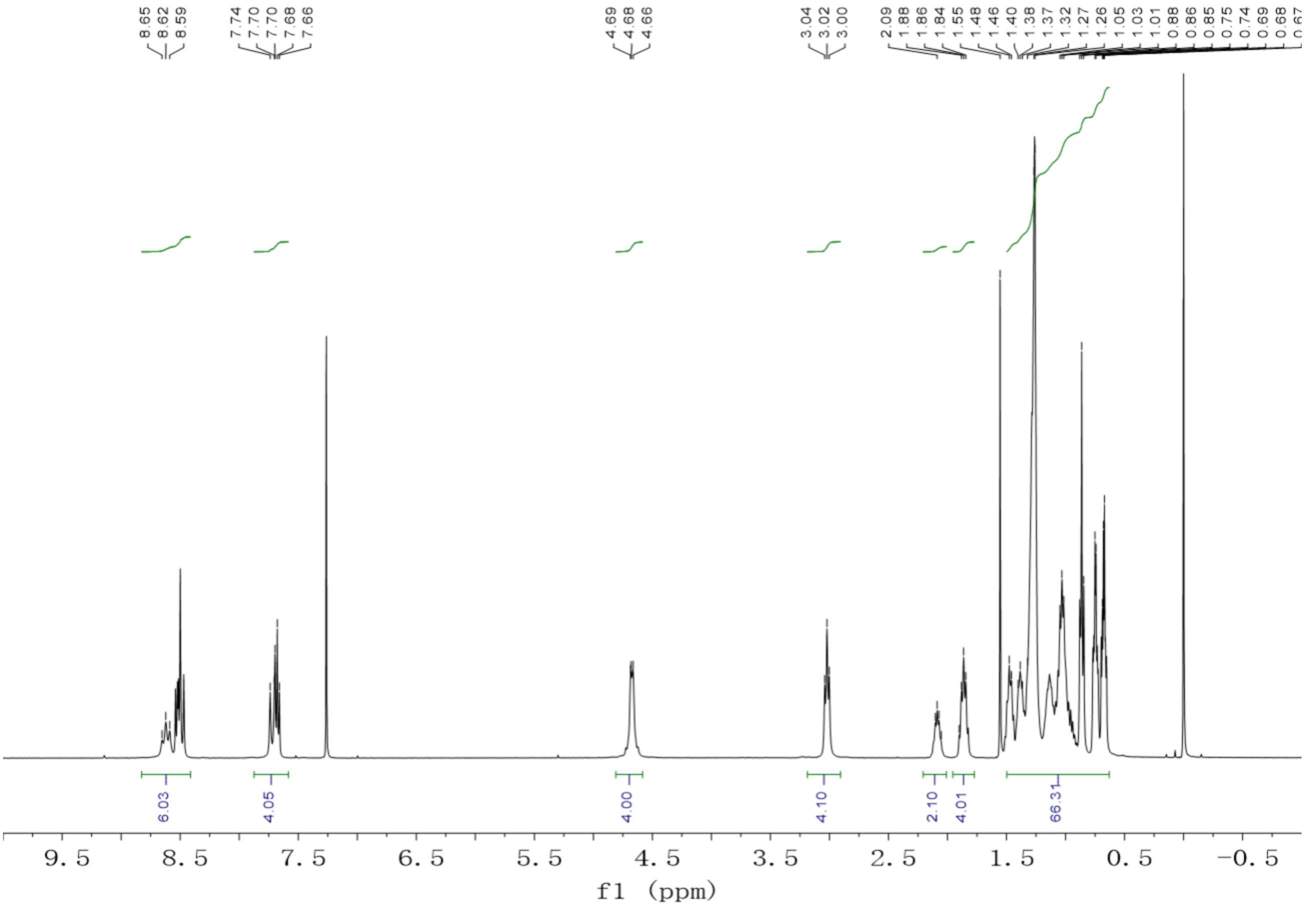

Supplementary Fig. 20. ^{1}HNMR spectra of BTPV-4F.

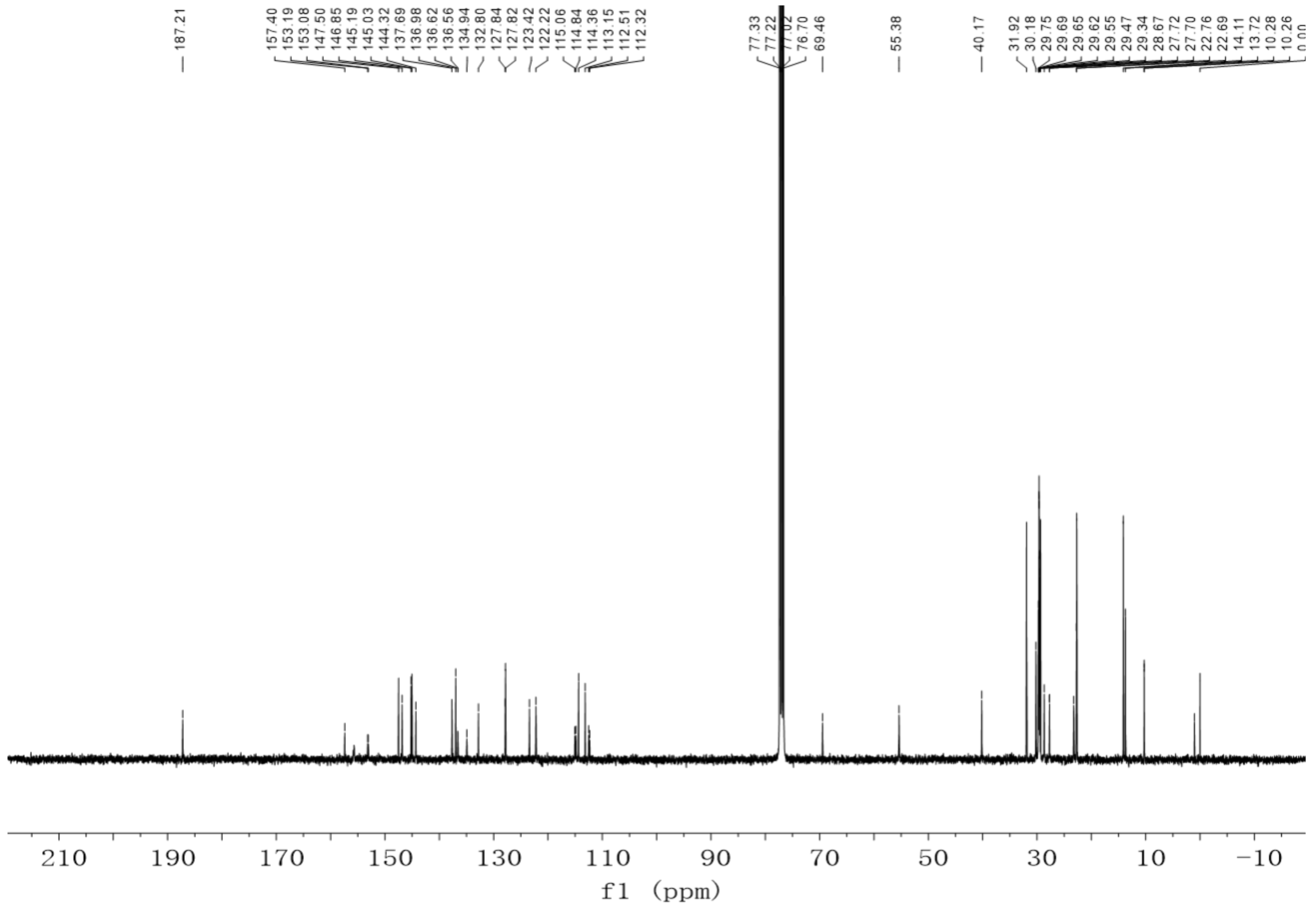

Supplementary Fig. 21. ^{13}CNMR spectra of BTPV-4F.

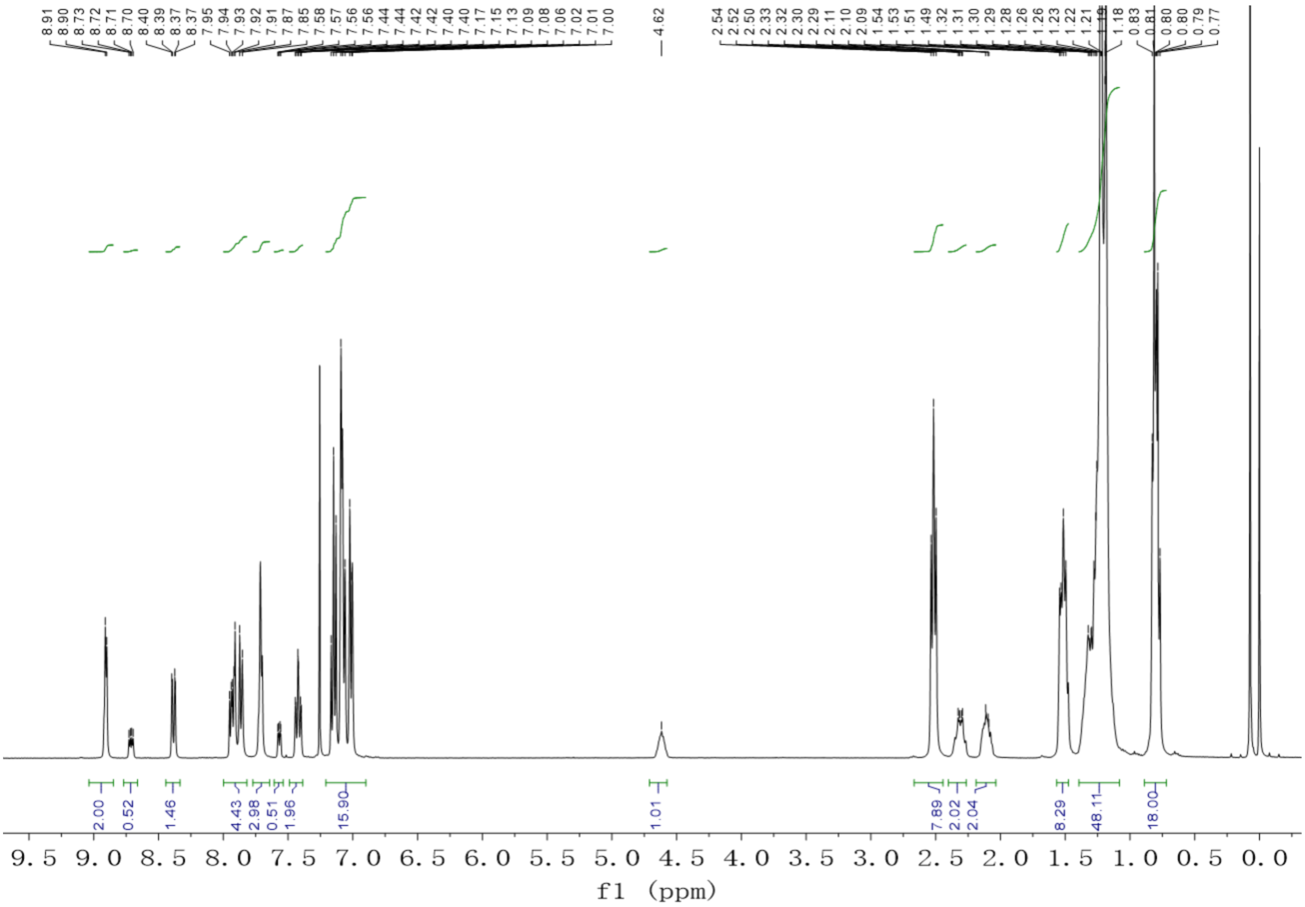

Supplementary Fig. 22. ^{1}HNMR spectra of *m*-DTC-2F.

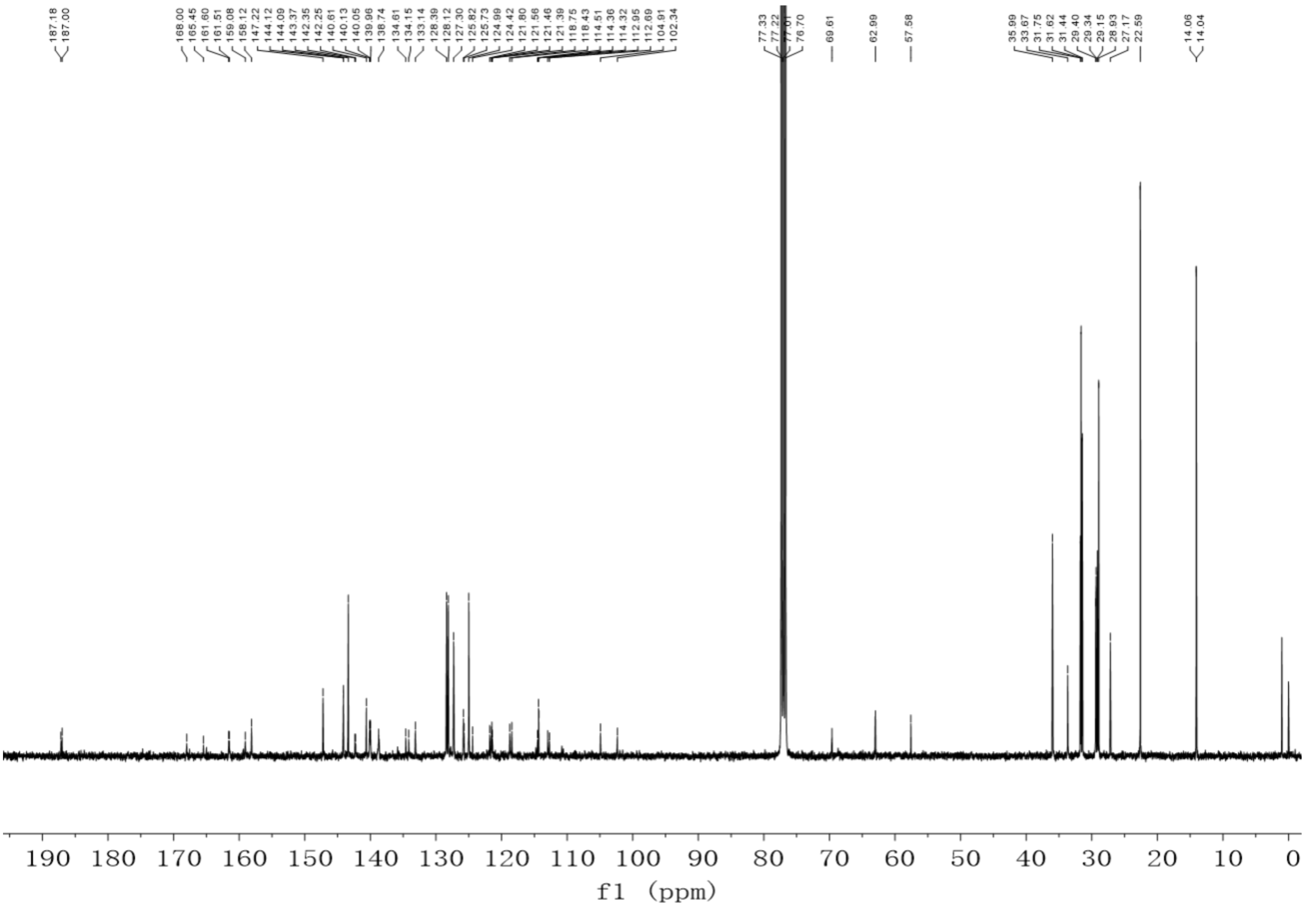

Supplementary Fig. 23. ^{13}CNMR spectra of *m*-DTC-2F.

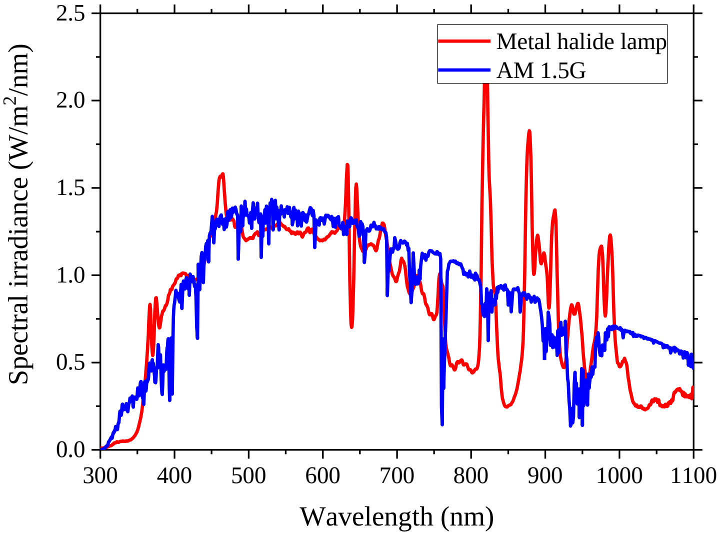

Supplementary Fig. 24. Illumination spectrum of light source used in *J*-*V* curve measurements.

SUPPLEMENTARY TABLES

Supplementary Table 1. Physicochemical properties and electronic energy levels of acceptors.

	λ_{max}[a] nm	λ_{edge}[a] nm	E_g^{opt}[b] eV	E_{HOMO}[c] eV	E_{LUMO}[c] eV
BTPV-4F	887	1021	1.21	-5.39	-4.08
m-DTC-2F	705	770	1.61	-5.67	-3.89

[a]Absorption of the films. [b] Optical bandgap calculated from the absorption edge of the films: E_g^{opt} = 1240/λ_{edge}. [c] Energy levels calculated according to the equation $E_{LUMO/HOMO} = -e\,(E_{red/ox} + 4.36)$ (eV)

Supplementary Table 2. Photovoltaic performance parameters of the OSCs based on PTB7-Th:BTPV-4F with different D:A ratio, with 0.5 % 1-CN as additive and thermal annealing at 100 °C for 5 min, under the illumination of AM1.5G, 100 mW cm^{-2}.

D/ A ratios	V_{oc} (V)	J_{sc} (mA cm^{-2})	FF (%)	PCE (%)
1:1	0.65	27.2	65.3	11.5
1:1.5	0.65	28.3	65.9	12.1
1:1.7	0.64	27.7	65.7	11.6

Supplementary Table 3. Photovoltaic performance parameters of the ternary OSCs based on PTB7-Th:BTPV-4F:$PC_{71}BM$ with different $PC_{71}BM$ content, and with 0.5 % 1-CN as additive and thermal annealing at 100 °C for 5 min, under the illumination of AM1.5G, 100 mW cm^{-2}.

$D/A_1/A_2$ ratios	V_{oc} (V)	J_{sc} (mA cm^{-2})	FF (%)	PCE (%)
1:1.5:0.1	0.66	28.5	67.6	12.7
1:1.5:0.15	0.67	28.9	69.3	13.4
1:1.5:0.20	0.67	27.7	69.5	12.9

Supplementary Table 4. Photovoltaic performance parameters of the OSCs based on PTB7-Th:BTPV-4F:$PC_{71}BM$ with different CN additive volume ratio, with D/A weight ratio of 1:1.5:0.15 and thermal annealing at 100 °C for 5 min, under the illumination of AM1.5G, 100 mW cm^{-2}.

CN (vol%)	V_{oc} (V)	J_{sc} (mA cm^{-2})	FF (%)	PCE (%)
0	0.68	20.9	61.4	8.7
0.2	0.68	25.6	65.6	11.4
0.5	0.67	28.9	69.3	13.4
0.8	0.66	28.5	68.2	12.8

Supplementary Table 5. Photovoltaic performance parameters of the OSCs based on PTB7-Th:BTPV-4F:$PC_{71}BM$ with different annealing temperature, and with D/A weight ratio of 1:1.5:0.15 and thermal annealing at 100 °C for 5 min, under the illumination of AM1.5G, 100 mW cm^{-2}.

Annealing temperature (°C)	V_{oc} (V)	J_{sc} (mA cm^{-2})	FF (%)	PCE (%)
As cast	0.68	26.2	66.1	11.8
100	0.67	28,5	68.6	13.1
110	0.67	28.9	69.3	13.4
120	0.66	28.2	68.9	12.8

Supplementary Table 6. Photovoltaic performance parameters of the OSCs based on PTB7-Th:BTPV-4F:$PC_{71}BM$ with different active layer thickness, under the illumination of AM1.5G, 100 mW cm^{-2}.

Thickness (nm)	V_{oc} (V)	J_{sc} (mA cm^{-2})	FF (%)	PCE (%)
80	0.67	27.2	69.7	12.7
100	0.67	28.9	69.3	13.4
120	0.66	27.8	66.7	12.2

Supplementary Table 7. Survey of the optical bandgap of the narrow bandgap acceptors and device performance of the acceptors-based OSCs reported in literatures.

Active layer	Optical bandgap (eV)	V_{oc} (V)	J_{sc} (mA cm^{-2})	$J_{sc, EQE}$ (mA cm^{-2})	FF (%)	PCE (%)	Ref.
PTB7-Th:COi8DFIC:PC$_{71}$BM	1.26	0.70	28.20	26.92	71.0	14.08	1
PTB7-Th:F8IC	1.27	0.67	25.61	24.64	71.5	12.30	2
PTB7-Th:FOIC	1.32	0.74	24.0	23.1	67.1	12.00	3
PTB7-Th:IEICO-4F	1.24	0.71	24.90	24.00	66.3	10.4	4
PM6:Y1-4F	1.31	0.838	24.8	24.1	71.2	14.80	5
PM6:Y11	1.31	0.85	26.56	24.15	73.1	16.54	6
PTB7-Th:IEICO-4Cl	1.23	0.73	22.80	22.20	62.0	10.30	7
PTB7-Th:3TT-OCIC	1.29	0.69	27.60	26.02	69.0	13.13	8
PTB7-Th:3TT-FIC	1.25	0.67	27.73	26.41	73.0	13.53	9
PTB7-Th:P6IC	1.30	0.69	25.0	23.7	70.2	10.2	10
PTB7-Th:IXIC-2Cl	1.30	0.73	23.6	23.3	70.9	12.2	11
PTB7-Th:IXIC-4Cl	1.25	0.69	22.9	22.8	71.2	10.2	11
PTB7-Th:IFIC-i-4F	1.27	0.65	24.85	24.01	67.0	10.87	12
PTB7-Th:DTPC-DFIC	1.22	0.76	21.92	21.27	61.3	10.21	13
PTB7-Th:FUIC	1.22	0.69	22.9	22.1	70.6	11.20	14
PBDB-T:H2	1.23	0.781	24.40	23.50	69	13.15	15
PBDB-T:H3	1.23	0.757	25.84	25.78	70	13.75	15
**This work**	1.21	0.67	28.9	28.3	69.3	13.4	

Supplementary Table 8. Crystallographic Parameters from GIWAX measurement.

Active layer	π-π stacking distance(OP)		π-π stacking coherence (OP)		Integrated Intensity
	q (Å^{-1})	d (Å)	Δq (Å^{-1})	CCL [010] Å	
PTB7-Th:BTPV-4F	1.76	3.57	0.15	41	1.42×10^{-7}
PTB7-Th:BTPV-4F:$PC_{71}BM$	1.77	3.55	0.14	46	1.71×10^{-7}

Note: The (010) π-π stacking peaks of PTB7-Th and BTPV-4F are relatively close together and the disorder is so large that the contributions in the blends cannot be separated.

Supplementary Table 9. Photovoltaic performance parameters of the OSCs based on PM6:*m*-DTC-2F with different D:A ratio, and with 0.3 % DIO as additive and thermal annealing at 120 °C for 5 min, under the illumination of AM1.5G, 100 mW cm^{-2}.

D/A ratios	V_{oc} (V)	J_{sc} (mA cm^{-2})	FF (%)	PCE (%)
1:1	1.00	16.3	69.5	11.3
1:1.5	1.00	17.1	71.3	12.2
1:1.7	0.99	16.8	70.7	11.8

Supplementary Table 10. Photovoltaic performance parameters of the OSCs based on PM6:*m*-DTC-2F with different DIO additive volume, and with D/A weight ratio of 1:1.5 and thermal annealing at 120 °C for 5 min, under the illumination of AM1.5G, 100 mW cm^{-2}.

DIO (vol%)	V_{oc} (V)	J_{sc} (mA cm^{-2})	FF (%)	PCE (%)
0	1.01	16.4	69.5	11.5
0.1	1.00	16.7	69.7	11.7
0.3	1.00	17.1	71.3	12.2
0.5	0.99	17.2	70.7	12.0

Supplementary Table 11. Photovoltaic performance parameters of the OSCs based on PM6:*m*-DTC-2F with different annealing temperature and with D/A weight ratio of 1:1.5 and thermal annealing for 5 min, under the illumination of AM1.5G, 100 mW cm^{-2}.

Anneling temperature (°C)	V_{oc} (V)	J_{sc} (mA cm^{-2})	FF (%)	PCE (%)
As cast	1.01	16.0	68.7	11.1
100	1.00	16.8	70.5	11.8
120	1.00	17.1	71.3	12.2
140	0.99	16.8	69.4	11.5

Supplementary Table 12. Photovoltaic performance parameters of the OSCs based on PM6:*m*-DTC-2F with different active layer thickness, under the illumination of AM1.5G, 100 mW cm^{-2}.

Thickness (nm)	V_{oc} (V)	J_{sc} (mA cm^{-2})	FF (%)	PCE (%)
100	1.00	17.1	71.3	12.2
120	1.00	17.3	69.8	12.1
140	0.99	17.4	68.3	11.7
160	0.98	16.9	67.5	11.2

Supplementary Table 13. Photovoltaic performance parameters of the tandem OSCs with different thicknesses of the sub-cells, under the illumination of AM1.5G, 100 mW cm^{-2}.

Thickness (nm)		V_{OC}	J_{SC}	FF	PCE
Front cell	Rear cell	(V)	(mA cm^{-2})	(%)	(%)
120	100	1.66	13.2	70.0	15.6
130	100	1.65	13.7	68.6	15.8
140	100	1.65	14.3	67.7	16.4
150	100	1.64	14.1	67.2	15.9
160	100	1.64	14.0	66.7	15.6
140	80	1.65	13.8	68.5	15.8
140	120	1.64	14.0	67.0	15.7

SUPPLEMENTARY METHOD

DFT calculation

ORCA (version 4.2.1) was utilized for all the DFT calculations mentioned in this article[16]. The molecular geometries were optimized with a range-separated hybrid functional of ωB97X-D3 and a basis set of def2-SVP. The straight and branched alkyl chains were simplified to methyl and isopropyl, respectively, for saving time. The single point energies and gradients were further calculated with a perturbatively corrected double-hybrid functional of PWPB95 with a dispersion correction of D3BJ and a basis set of def2-TZVPP[17,18]. The band gaps were calculated with a functional of B3PW and a basis set of def2-TZVP. The excited states incorporating with SMD solvation model were calculated by TD-DFT with a hybrid functional of B3LYP and a basis set of def2-SVP[19]. The localized orbital locator (LOL), Mayer bond order were analyzed by Multiwfn and VMD (version 1.9.3) for visualization[20].

Theoretical efficiency calculation

For the 2-terminal monolithic tandem cell with two sub-cells in series, The PCE of the tandem cell is therefore calculated as follows:

$$\mathrm{PCE}_{\mathrm{Tandem}} = V_{\mathrm{OC\ Tandem}} \times J_{\mathrm{SC\ Tandem}} \times \mathrm{FF}_{\mathrm{Tandem}} / P_{\mathrm{light}}$$

The three photovoltaic parameters V_{oc}, J_{sc} and FF of a 2-terminal monolithic tandem cell are obtained as follows:

1. The V_{oc} of sub-cells is determined by the optical bandgap (E_g) and V_{oc} loss (E_{loss}) of the sub-cells.

$$V_{\mathrm{oc,\ front}} = E_{\mathrm{g,\ front}} - E_{\mathrm{loss,\ front}}$$

$$V_{\mathrm{oc,\ rear}} = E_{\mathrm{g,\ rear}} - E_{\mathrm{loss,\ rear}}$$

For the E_{loss} of sub-cells, the $E_{\mathrm{loss,\ front}}$ and $E_{\mathrm{loss,\ rear}}$ are assumed to be 0.55eV according to reported works.

The V_{oc} of the tandem cell is therefore calculated as being the exact sum of the respective V_{oc} of each sub-cell are assumed to be

$$V_{oc,\ tandem} = V_{oc,\ front} + V_{oc,\ rear}$$

2. The J_{sc} of the tandem cell could be obtained from equation

$$J_{sc,\ tandem} = \min(J_{sc,\ front}, J_{sc,\ rear})$$

The J_{sc} of the front cell in the tandem device under AM1.5G is given by

$$\int_{300}^{\lambda_1} Sun(\lambda) \cdot EQE(\lambda) \cdot d\lambda$$

where λ_1 is the absorption onset ($1240/E_{g,\ front}$) of front cell, $Sun(\lambda)$ is the spectral irradiance in AM 1.5G, The EQE(λ) is assumed as 75% with all wavelengths.

The J_{sc} of the rear cell in the tandem device under AM1.5G is given by

$$\frac{1}{2}\int_{300}^{\lambda_2} Sun(\lambda) \cdot EQE(\lambda) \cdot d\lambda$$

where λ_2 is the absorption onset ($1240/E_{g,\ rear}$) of rear cell, $Sun(\lambda)$ is the spectral irradiance in AM 1.5G, The EQE(λ) is assumed as 75% with all wavelengths.

3. The FF of the tandem cell is assumed to be 0.75.

Supplementary References

1. Xiao, Z. et al. Ternary organic solar cells offer 14% power conversion efficiency. *Sci. Bull,* **62,** 1562–1564 (2017)
2. Dai, S. *et al.* Enhancing the Performance of Polymer Solar Cells via Core Engineering of NIR-Absorbing Electron Acceptors. *Adv. Mater.* **30,** 1706571 (2018).
3. Li, T. *et al.* Fused tris(thienothiophene)-based electron acceptor with strong near-infrared absorption for high-performance As-Cast Solar Cells. *Adv. Mater.* **30,** 1705969 (2018).
4. Yao, H. *et al.* Design, Synthesis, and Photovoltaic Characterization of a Small Molecular Acceptor with an Ultra-Narrow Band Gap. *Angew. Chem. Int. Ed.* **56,** 3045–3049 (2017).
5. Wang, R. *et al.* Rational Tuning of Molecular Interaction and Energy Level Alignment Enables High-Performance Organic Photovoltaics. *Adv. Mater.* **31,** 1904215 (2019).
6. Liu, S. *et al.* High-efficiency organic solar cells with low non-radiative recombination loss and low energetic disorder. *Nat. Photonics* 1–11 (2020). doi:10.1038/s41566-019-0573-5
7. Cui, Y. *et al.* Efficient Semitransparent Organic Solar Cells with Tunable Color enabled by an Ultralow-Bandgap Nonfullerene Acceptor. *Adv. Mater.* **29,** 1703080 (2017).
8. Gao, H. H. *et al.* Achieving Both Enhanced Voltage and Current through Fine-Tuning Molecular Backbone and Morphology Control in Organic Solar Cells. *Adv. Energy Mater.* **9,** 1901024 (2019).
9. Gao, H. H. *et al.* A New Nonfullerene Acceptor with Near Infrared Absorption for High Performance Ternary-Blend Organic Solar Cells with Efficiency over 13%. *Adv. Sci.* **5,** 1800307 (2018).
10. Ma, W. *et al.* Enhancing Performance of Fused-Ring Electron Acceptor Using Pyrrole Instead of Thiophene. *ACS Appl. Mater. Interfaces* **12**, 14029-14036 (2020).
11. Chen, Y. *et al.* Modulation of End Groups for Low-Bandgap Nonfullerene Acceptors Enabling High-Performance Organic Solar Cells. *Adv. Energy Mater.* **8,** 1801203 (2018).
12. Chen, F. X. *et al.* Near-Infrared Electron Acceptors with Fluorinated Regioisomeric Backbone for Highly Efficient Polymer Solar Cells. *Adv. Mater.* **30,** 1803769 (2018).
13. Yao, Z. *et al.* Dithienopicenocarbazole-Based Acceptors for Efficient Organic Solar Cells with

Optoelectronic Response Over 1000 nm and an Extremely Low Energy Loss. *J. Am. Chem. Soc.* **140,** 2054–2057 (2018).

14. Li, T. *et al.* Facile synthesis of high-performance nonfullerene acceptor isomers via a one stone two birds strategy. *J. Mater. Chem. A* **7,** 20667–20674 (2019).

15. He, C. *et al.* Near infrared electron acceptors with a photoresponse beyond 1000 nm for highly efficient organic solar cells. *J. Mater. Chem. A* **8**, 18154-18161 (2020).

16. Neese, F. Software update: the ORCA program system, version 4.0. *Wiley Interdisciplinary Reviews: Computational Molecular Science* **8,** 33 (2018).

17. Grimme, S., Antony, J., Ehrlich, S. & Krieg, H. A consistent and accurate ab initio parametrization of density functional dispersion correction (DFT-D) for the 94 elements H-Pu. *The Journal of Chemical Physics* **132,** 154104 (2010).

18. Grimme, S., Ehrlich, S. & Goerigk, L. Effect of the damping function in dispersion corrected density functional theory. *Journal of Computational Chemistry* **32,** 1456–1465 (2011).

19. Marenich, A. V. et al. Universal solvation model based on solute electron density and on a continuum model of the solvent defined by the bulk dielectric constant and atomic surface tensions. *J. Phy. Chem. B* **113**, 6378-6396 (2017)

20. Lu, T. & Chen, F. Multiwfn: A multifunctional wavefunction analyzer. *Journal of Computational Chemistry* **33,** 580–592 (2012).
