# Supplementary material for: High performance tandem organic solar cells via a strongly infrared-absorbing narrow bandgap acceptor
Source: Nat Commun. 2021 Jan 8;12:178. doi: 10.1038/s41467-020-20431-6 (PMC7794321; doi:10.1038/s41467-020-20431-6)
Supplement: Supplementary file 3 — Reporting Summary [file 41467_2020_20431_MOESM3_ESM.pdf]

nature research

Corresponding author(s): Lei Meng; Yongfang Li; Harald Ade

Solar Cells Reporting Summary

Nature Research wishes to improve the reproducibility of the work that we publish. This form is intended for publication with all accepted papers reporting the characterization of photovoltaic devices and provides structure for consistency and transparency in reporting. Some list items might not apply to an individual manuscript, but all fields must be completed for clarity.

For further information on Nature Research policies, including our data availability policy, see Authors & Referees.

Experimental design

Please check: are the following details reported in the manuscript?

1. Dimensions

Item	Yes	No	Details
Area of the tested solar cells	☒	☐	Area of the tested solar cells is 0.05 cm2 defined by optical microscope (Olympus BX51).
Method used to determine the device area	☒	☐	Device area is determined by a mask with 0.048 cm2

2. Current-voltage characterization

Item	Yes	No	Details
Current density-voltage (J-V) plots in both forward and backward direction	☐	☒	Generally, organic photovoltaic devices do not have forward and backward problems. And we only scan the device in forward direction.
Voltage scan conditions<br>*For instance: scan direction, speed, dwell times*	☒	☐	The voltage was scanned from -1.5 V to 2 V. The voltage step and delay time were 10 mV and 1ms, respectively.
Test environment<br>*For instance: characterization temperature, in air or in glove box*	☒	☐	Devices were characterized at room temperature in In N2-filled glove box
Protocol for preconditioning of the device before its characterization	☐	☒	No preconditioning protocol.
Stability of the J-V characteristic<br>*Verified with time evolution of the maximum power point or with the photocurrent at maximum power point; see ref. 7 for details.*	☐	☒	We only tested the long-term stability.

3. Hysteresis or any other unusual behaviour

Item	Yes	No	Details
Description of the unusual behaviour observed during the characterization	☐	☒	No hysteresis was observed in our device.
Related experimental data	☐	☒	No.

4. Efficiency

Item	Yes	No	Details
External quantum efficiency (EQE) or incident photons to current efficiency (IPCE)	☒	☐	EQE curve is shown in Figure 2c and Figure 4d.
A comparison between the integrated response under the standard reference spectrum and the response measure under the simulator	☒	☐	Relative information is provided in Table 1-2. The integrated Jsc obtained from the EQE spectra agrees well with the Jsc value obtained from the J–V curves under the simulator within 2% deviation
For tandem solar cells, the bias illumination and bias voltage used for each subcell	☒	☐	Light bias obtained by 550 nm short wave pass filters and 850 nm long wave pass filters were selected to excite (saturate) the front and rear cells, respectively.

5. Calibration

Item	Yes	No	Details
Light source and reference cell or sensor used for the characterization	☒	☐	Relative information is provided in method section.
Confirmation that the reference cell was calibrated and certified	☒	☐	Relative information is provided in method section.

Calculation of spectral mismatch between the reference cell and the devices under test

☒ Yes ☐ No — Relative information is provided in method section.

6. Mask/aperture

Size of the mask/aperture used during testing

☒ Yes ☐ No — Device area is determined by a mask with area of 0.048 cm2.

Variation of the measured short-circuit current density with the mask/aperture area

☒ Yes ☐ No — The variation is within 0.3%.

7. Performance certification

Identity of the independent certification laboratory that confirmed the photovoltaic performance

☐ Yes ☒ No — The photovoltaic performance of our devices was not confirmed from independent certification laboratories

A copy of any certificate(s)
Provide in Supplementary Information

☐ Yes ☒ No — The photovoltaic performance of our devices was not confirmed from independent certification laboratories

8. Statistics

Number of solar cells tested

☒ Yes ☐ No — The average PCE of OSC is obtained from 30 independent devices.

Statistical analysis of the device performance

☒ Yes ☐ No — Statistical results of the devices are shown in figure 4e.

9. Long-term stability analysis

Type of analysis, bias conditions and environmental conditions
For instance: illumination type, temperature, atmosphere humidity, encapsulation method, preconditioning temperature

☒ Yes ☐ No — Long-term photostability analysis can be found in figure 5b and conditions can be found in method section.
